# Mindful Walking in Patients with Chronic Low Back Pain: A Randomized Controlled Trial

**DOI:** 10.1089/jicm.2021.0361

**Published:** 2022-06-07

**Authors:** Gabriele Rotter, Miriam Ortiz, Sylvia Binting, Juliane Tomzik, Frauke Reese, Stephanie Roll, Benno Brinkhaus, Michael Teut

**Affiliations:** Institute of Social Medicine, Epidemiology and Health Economics, Charité – Universitätsmedizin Berlin, corporate member of Freie Universität Berlin and Humboldt – Universität zu Berlin, Berlin, Germany.

**Keywords:** low back pain, complementary medicine, integrative medicine, randomized controlled trial

## Abstract

**Aim:**

The objective of this study was to investigate the effectiveness of a mindful walking program (MWP) in patients with chronic low back pain (CLBP).

**Methods:**

The trial was a two-armed, randomized, controlled single-center open clinical trial. The study was performed in the Outpatient Clinic for Integrative Medicine of the Charité–Universitätsmedizin Berlin. The participants were adults aged 18–65 years with CLBP (≥3 months) and an average low back pain within the past 7 days measured on a visual analog scale (VAS, 0 = no pain, 100 = worst imaginable pain) of at least 40 mm. The patients received either eight weekly MWP sessions or no intervention (control). The primary outcome was the perceived pain intensity assessed with a VAS (0–100 mm) after 8 weeks. The secondary outcomes included back function assessed by the Hannover Functional Questionnaire Backache (FFbH-R) and perceived stress assessed by the 14-item Cohen's Perceived Stress Scale (PSS-14). The results were obtained by analysis of covariance adjusted for the respective baseline values.

**Results:**

In total, 55 patients were randomized (MWP: *n* = 29, 82.8% female, mean (±standard deviation) age: 52.5 ± 8.6 years, pain: 56.4 ± 14.1 mm; control: *n* = 26, 84.6% female, 54.8 ± 7.5 years, pain: 55.4 ± 13.1 mm). After 8 weeks, compared with the control conditions, the MWP was not associated with a statistically significant benefit for pain (VAS), adjusted mean − 9.6 [−22.3 to 3.1], *p* = 0.136, clinical benefits for back function (FFbH-R), adjusted mean 2.2 [−4.2 to 8.6], *p* = 0.493, or stress (PSS-14), adjusted mean − 1.6 [−4.8 to 1.6], *p* = 0.326.

**Conclusion:**

In conclusion, compared with no intervention, mindful walking did not significantly improve pain, back function, or perceived stress in patients with CLBP.

**Clinical Trial registration:**

ClinicalTrials.gov (NCT01893073).

## Introduction

Low back pain is one of the leading causes of ill health globally and leads to a high disease burden.^1,2^ In Germany, the 3-month prevalence was reported at 25%,^3^ and the lifetime prevalence was between 74% and 85%.^4^ Not all patients with low back pain are sufficiently treated and continue to have relevant symptoms. The prevalence of nonspecific chronic low back pain (CLBP) was internationally reported to be between 4% and 20%.^5^

The usual CLBP treatment consists of a multimodal regimen, including nonsteroidal analgesics, educational interventions, relaxation therapy, and exercises such as walking and mind–body interventions.^6–8^ However, long-term treatment with analgesics might result in relevant adverse events, and there is growing interest in nonpharmacological simple and cost-effective therapies, including treatments originating from complementary and integrative medicine, such as mindful walking.^9–12^

Mindful walking combines walking as a low-intensity exercise with mindfulness training. The effectiveness of exercise and especially walking as a low-intensity exercise in the treatment of CLBP is well established.^7^ A meta-analysis of 17 studies investigating walking in patients with chronic musculoskeletal pain, including five studies investigating CLBP, found that walking can be an effective form of exercise or activity for individuals with CLBP.^13^

Mindfulness training is a mental stress reduction strategy derived from Buddhist meditation. Mindfulness is defined as the tendency to encounter moment-to-moment experiences without being lost in unhelpful or distressing thoughts triggered by the experience.^14^ Being mindful is also often related to be open, nonjudgmental, friendly, curious, accepting, compassionate, and kind.^15^ Mindfulness-based interventions have been reported to be effective in alleviating various bio-psychosocially influenced conditions, including pain.^16^

The most commonly studied mindfulness training program is mindfulness-based stress reduction (MBSR). In various pain conditions, including low back pain, pain-relieving effects and strengthening of coping skills with MBSR have been reported.^17–19^ Psychological factors, such as anxiety, stress, catastrophizing pain, and lack of coping strategies, play an important role in causing or aggravating CLBP. In therapy, it is important to also address the psychological sphere. Reviews and systematic reviews report mindful interventions' beneficial impact.^16,20,21^

A systematic review and meta-analysis of 30 randomized controlled trials (RCTs) investigating chronic pain showed improvement in chronic pain management after mindfulness meditation interventions.^20^ The approach of mindful walking is of great interest, and patients reported a broad range of perceived benefits in a recently published qualitative study.^22^

The rationale for this trial was that a combination of mindfulness and mental stress reduction strategies with exercise would provide a useful treatment strategy for CLBP. Therefore, we developed an easy-to-follow training program that combines mindfulness training with walking exercise in a mindful walking program (MWP). An additional rationale for the development and testing of the MWP is that such a program can simultaneously address the following two mechanisms linked to pain perception: physical activity and stress/distress.

Individuals have competing demands for time (i.e., people are busy, and it can be difficult to meet both physical activity and mental health self-care recommendations with limited time); a program that may offer benefits for physical activity, stress, and pain simultaneously offers significant utility and future promise.

The aim of this RCT was to investigate the effectiveness of mindful walking in patients with CLBP.

## Methods

### Study design and setting

In a single-center open two-armed randomized controlled clinical trial, CLBP patients were randomized to either an MWP group or no study intervention (control group [CG]). The randomization (1:1 ratio, consecutive ID numbers) was generated by a data manager as a computer-generated randomization sequence by using SAS 9.3 software.^23^ The sequence was concealed by use of a computer interface implemented in the electronic case report form and kept by the study nurse. The study physician contacted the study center (study nurse) by telephone and provided the participants' inclusion information.

After entering these data, the participants were assigned to the intervention or CG, and the results of the randomization were reported to the study physician. The study was performed at the Outpatient Clinic for Integrative Medicine of the Charité–Universitätsmedizin in Berlin, Germany. This study followed the standards of the Declaration of Helsinki^24^ and the ICH-GCP guidelines.^25^ All patients gave oral and written informed consent before study inclusion. The study was approved by the Ethics Committee of the Charité–Universitätsmedizin, Berlin, Germany (EA1/118/13) and was registered at ClinicalTrials.gov.

### Patients

Patients were recruited by local daily newspapers and the website of the outpatient department for integrative medicine, Charité. Patients between 18 and 65 years of age with a clinical diagnosis of CLBP (disease duration of at least 3 months) and an average low back pain within the past 7 days measured on a visual analog scale (“VAS pain,” 0–100 mm; 0 = no pain, 100 = worst imaginable pain)^26^ of at least 40 mm were included.

The exclusion criteria were active walking or jogging (past 6 weeks, more than 60 min/week); regular meditation, relaxation exercise, or mindfulness exercises (past 6 weeks, more than 30 min/week); complementary medicine or use of other nonpharmacological therapies; participation in another clinical trial (past 3 months); neurological symptoms related to the spine; elevated risk of falls or inability to walk; angina pectoris (past 3 months); chronic respiratory disease with respiratory insufficiency; intake of central nervous system-acting analgesics (such as opioids, past 6 weeks); known renal and/or hepatic disease; severe organic, psychological, or psychiatric disorders not permitting study participation; and ongoing application for early retirement due to low back pain.

### Study interventions

The MWP was based on our previous study in patients with stress symptoms,^27^ with an adjustment toward more physically active exercise. Over the course of 8 weeks, the patients allocated to the MWP group participated in eight group sessions, each restricted to 15 participants and lasting 50 to 60 min. The instructions regarding the mindful aspect included health education and an explanation of the term “mindfulness” and essential aspects of mindfulness awareness.

The instructions regarding the mindful aspect included an explanation of the term and essential aspects of mindfulness. At the beginning, the essential components of active walking in the sense of “good mood walking” were taught, and individual techniques of walking were instructed (rolling of the foot, arm–shoulder movement, and torso posture). In the following walking lessons, repetitions of the concept of mindfulness occurred, and the technique of mindful walking was practiced in the sense of a guided and feedback-controlled learning process.

In the first week, the focus was on the conscious rolling of the feet; in the second week, the swinging of the arms was additionally learned; and in the third week, the conscious torso posture was taught. The last week was dedicated to the complex implementation of all learned elements. In this intervention program, the main task was the conscious awareness of one's own body and thoughts. The participants were instructed to focus their awareness on the regions and movements introduced in each session. In addition, awareness of breathing while walking was taught. Strict adherence to the prescribed technique was not necessary, but the focus was on trying and adapting to individual physical conditions.

In addition, the participants were encouraged to be mindful of themselves and their environment, including the people in their surroundings, in their daily lives and were asked to report their experiences with practicing mindfulness in each session (Table 1). The patients in the MWP group were advised and instructed to self-exercise between group sessions. The MWP was carried out by a physiotherapist and physician and a sports therapist. All were trained in MBSR techniques, and the trainers received additional instructions from the principal investigator. Because the objective was to investigate the effectiveness,^28^ the patients in the CG received no study intervention but could participate in eight, free-of-cost MWP sessions after study completion outside of the study.

**Table 1.** Structure of a Mindful Walking Session (50 to 60 Min)

5 min	Group meets in a park surrounding; greetings (“Tiergarten, Berlin”)
10 min	Stretching exercises to warm up and short walking instructions.
15–25 min	An increasingly active pace of walking with a “good mood walking” attitude with individual step length and foot roll intensity emphasizing an actively moving forward movement of the foot.
10 min	Individual mindful walking. Participants were instructed to mindfully observe and focus on their bodily sensations (foot movement, trunk erection and movement, arm-shoulder movement, breathing) while walking and maintaining focus on their moment-to-moment experiences without being lost in unhelpful or distressing thoughts triggered by the experience. If this was experienced as a problem, the participants were instructed to focus their awareness on their breath while inhaling and exhaling.
5 min	Stretching exercises.
5 min	A feedback round was used to share and discuss the experiences, followed by farewells.

The patients in both groups were allowed and instructed to use rescue medication on demand (paracetamol; maximum dosage, four times 500 mg/day).

### Outcome parameters and data collection

Data were collected at baseline and after 8 and 12 weeks by using standardized patient questionnaires.

The primary outcome was perceived low back pain intensity after 8 weeks; the patients rated pain over the previous week on the VAS (0–100 mm).^26,29^ The minimal clinically important difference (MCID) has been reported to be 15 mm.^30,31^ Low back pain by VAS after 12 weeks and all other outcomes were considered secondary outcomes. Back function was measured with the Hannover Functional Questionnaire Backache (FFbH-R; 0% = minimal functional capacity, 100% = maximal functional capacity; assumed MCID: 12%).^32^

Perceived stress was measured by the 14-item Cohen's Perceived Stress Scale (PSS-14; range: 0–56, with lower scores indicating a lower stress level; no MCID determined).^33^ Health-related quality of life was assessed by the Short-Form-36 Health Survey (SF-36)^34,35^ (assumed MCID: 5 points). The patients also rated the treatment expectancy at baseline if randomized to the MWP group or CG (“cure,” “significant recovery,” “slight recovery,” and “no recovery”). After 12 weeks, the patients rated the changes in their low back pain (“slightly reduced,” “significantly reduced,” “completely reduced,” “not changed,” and “worsened”). Safety (adverse events and serious adverse events) was assessed across the whole study period. The intake of paracetamol and all other analgesics during the first 8 weeks was documented in the patient diaries.

### Statistical analysis

The sample size calculation was based on the primary outcome (VAS pain after 8 weeks), with an assumed difference of 15 mm (MCID)^30,31^ between the treatment groups and an assumed common standard deviation (SD) of 15 mm. A two-sided *t*-test would have a power of 80% with a significance level of 5% if 24 patients were enrolled in each group (total *n* = 48), including a dropout rate of ∼25%. nQuery Advisor 6.02 was used for this calculation.

The primary analysis of the primary outcome was performed by using an analysis of covariance (ANCOVA) with a fixed-factor treatment group adjusted for the baseline VAS pain value. The significance level was 5%. Secondary outcomes were analyzed in a similar manner as the primary outcome, that is, by ANCOVA adjusted for the respective baseline values and were considered exploratory. The results are reported as adjusted group means with 95% confidence intervals (95% CI) and the *p*-value for the treatment group comparison. All tests and CIs were two sided. All data were analyzed based on the intention-to-treat principle by using the full analysis set with all available data without imputing missing data, based on the original assigned group. Adverse events are descriptively presented by frequency for each treatment group.

For the primary outcome, sensitivity analyses included the replacement of missing values using the last value carried forward method and ANCOVA adjusted for the baseline value, education, and duration of CLBP. In addition, an analysis for the primary outcome based on the per-protocol (PP) population excluded patients if at least one of the following criteria was met: not treated based on group allocation; fewer than six group sessions attended (MWP group only); and intake of analgesics other than paracetamol (MWP group and CG).

The analgesic costs were assessed by using the prices for daily defined dosages (DDD)^36^ provided by the Drug Prescription Report 2014. The DDD was calculated for the first 8 weeks. The overall analgesic costs were calculated by multiplying the DDD prices by the number of days the drug was taken. The statistical analyses were performed by using the software packages SAS 9.3^23^ and R, version 3.6.3.^37^

## Results

### Patients and study interventions

**FIG. 1.**
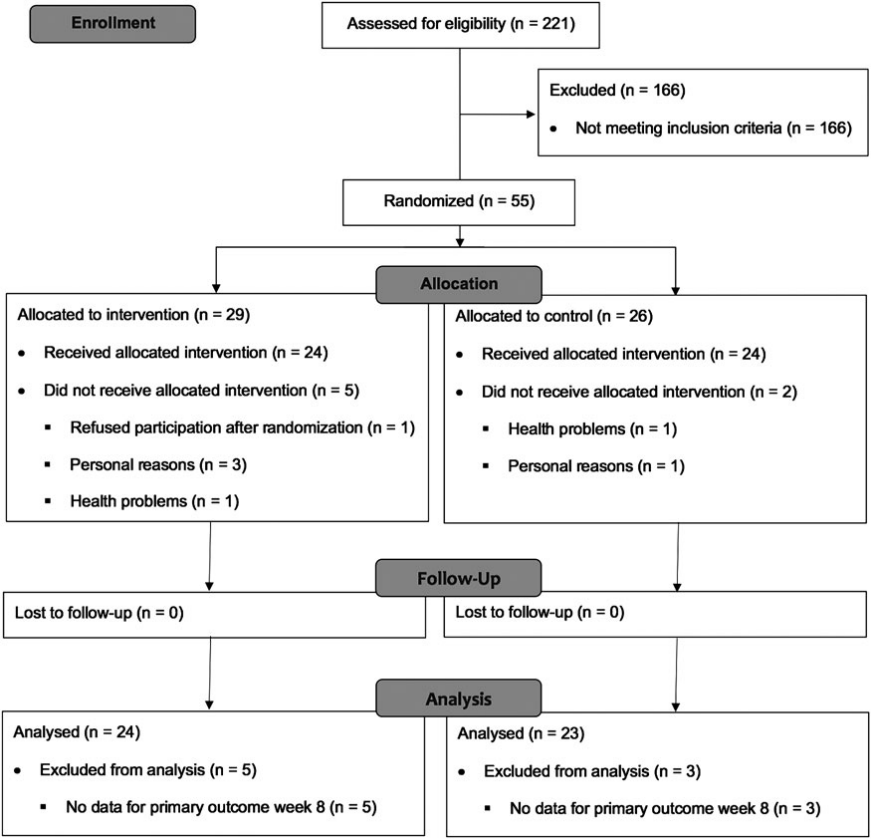
Flow chart. Recruitment, treatment and follow-up of patients.

Between May 2013 and October 2013, of the 221 screened patients, 55 patients were randomized (MWP group, *n* = 29; CG, *n* = 26). After randomization and before the first intervention, five (17.2%) patients randomized to the MWP group refused to participate and dropped out, and three (11.5%) patients randomized to the CG dropped out (Fig. 1). Five patients in the MWP group attended the course less than six times. One trainer conducted 80% of the courses.

Most patients were female, and the mean age was older than 50 years (MWP group: 82.8% female, mean ± SD: 52.5 ± 8.6 years; CG: 84.6% female, 54.8 ± 7.5 years; Table 2). There were relevant baseline differences between the groups for the following parameters: Patients allocated to the MWP group more often had a university entrance qualification (58.6% vs. 38.5%, respectively), and they had a longer duration of CLBP (8.4 ± 9.9 vs. 6.8 ± 6.2) years, than those allocated to the CG. The VAS scores for pain were comparable at 56.4 ± 14.1 versus 55.4 ± 13.1 mm in the two groups. Regarding treatment expectancy, most patients in both groups expected a “significant recovery” if randomized to the MWP (MWP group: 79.3%; CG: 69.2%) and “no recovery” if randomized to the control condition (MWP group: 86.2%; CG: 84.6%).

**Table 2.** Baseline Characteristics of Patients

	Mindful walking program* n* = 29 Mean ± SD/*n *(%)	Control* n* = 26 Mean ± SD/*n *(%)
Age, years	52.5 ± 8.7	54.8 ± 7.5
Sex, female	24 (82.8)	22 (84.6)
BMI, kg/m^2^	25.7 ± 4.4	26.2 ± 5.3
German university entrance qualification (Abitur)	17 (58.6)	10 (38.5)
Employed	19 (65.5)	16 (61.5)
Two-person household	16 (55.2)	12 (46.2)
Physically active	22 (75.9)	16 (61.5)
Duration of CLBP, years	9.6 ± 10.1	7.6 ± 10.1
VAS pain (0–100 mm)^a^	56.4 ± 14.1	55.4 ± 13.1
FFbH-R (0%–100%)^b^	70.7 ± 13.7	73.6 ± 17.5
PSS-14 (0–56)^a^	31.0 ± 7.0	30.9 ± 8.5
SF-36 physical component scale (0–100)^b^	41.1 ± 6.9	40.9 ± 8.0
SF-36 mental component scale (0–100)^b^	46.5 ± 9.8	45.4 ± 11.9
Intake of analgesics (last 4 weeks)	10 (34.5)	9 (34.6)
Among them intake of >1 analgesic	0	2 (7.7)
Analgesics (last 4 weeks)		
Paracetamol	1 (3.5)	2 (7.7)
Ibuprofen	5 (17.2)	7 (26.9)
Acetylsalicylic acid	2 (6.9)	1 (3.9)
Metamizole	1 (3.5)	1 (3.9)
Diclofenac	1 (3.5)	4 (15.4)
Patients' expectation if randomized to intervention		
Cure	0	3 (11.5)
Significant recovery	23 (79.3)	18 (69.2)
Slight recovery	6 (20.7)	5 (19.2)
No recovery	0	0
Patients' expectation if randomized to control		
Cure	0	0
Significant recovery	1 (3.4)	0
Slight recovery	3 (10.3)	4 (15.4)
No recovery	25 (86.2)	22 (84.6)

^a^ Lower values indicate better status.

^b^ Higher values indicate better status.

BMI, body mass index; CLBP, chronic low back pain; FFbH-R, Hannover functional questionnaire backache; PSS-14, 14-item Cohens' Perceived Stress Scale; SF-36, Short-Form-36 Health Survey; VAS, visual analog scale.

After 8 weeks, the between-group differences in the primary and all secondary outcomes tended to slightly favor the MWP group in comparison to the CG but failed to reach clinical relevance (Table 3). The primary outcome, low back pain assessed with the VAS, was not significantly different between the MWP group and the CG, with adjusted means [95% CI] of 35.9 [26.9–44.8] and 45.4 [36.3–54.5], respectively, and adjusted mean difference [95% CI] of −9.6 [−22.3 to 3.1], *p*-value = 0.136.

**Table 3.** Primary and Secondary Outcomes at Weeks 8 and 12

	n	MWP Adjusted mean, [95% CI]^a^	Control Adjusted mean, [95% CI]^a^	Mean difference (MWP – control) Adjusted mean, [95% CI]^a^	*p* ^a^
VAS pain (0–100 mm),^b^ MCID 15 mm (average neck pain during the previous 7 days)					
8 weeks (primary outcome)	47	35.9 [26.9 to 44.8]	45.4 [36.3 to 54.5]	−9.6 [−22.3 to 3.1]	0.136
12 weeks	47	34.8 [25.6 to 44.1]	47.4 [38.0 to 56.9]	−12.6 [−25.8 to 0.6]	0.061
FFbH-R (0–100%),^c^ assumed MCID 12%					
8 weeks	48	75.1 [70.6 to 79.7]	72.9 [68.4 to 77.5]	2.2 [−4.2 to 8.6]	0.493
12 weeks	47	74.9 [70.1 to 79.8]	74.0 [69.1 to 79.0]	0.9 [−6.0 to 7.9]	0.792
PSS-14 (0–56)^b^					
8 weeks	48	29.0 [26.7 to 31.2]	30.5 [28.3 to 32.8]	−1.6 [−4.8 to 1.6]	0.326
12 weeks	47	28.1 [25.6 to 30.7]	31.2 [28.6 to 33.8]	−3.1 [−6.7 to 0.6]	0.096
SF-36 physical component scale (0–100),^c^ assumed MCID 5 points					
8 weeks	48	43.4 [40.8 to 46.1]	39.9 [37.3 to 42.6]	3.5 [−0.2 to 7.3]	0.066
12 weeks	47	43.6 [40.9 to 46.3]	39.9 [37.1 to 42.6]	3.7 [−0.2 to 7.6]	0.059
SF-36 mental component scale (0–100),^c^ assumed MCID 5 points					
8 weeks	48	45.7 [42.7 to 48.7]	46.9 [43.9 to 49.9]	−1.2 [−5.5 to 3.0]	0.567
12 weeks	47	46.7 [42.7 to 50.6]	44.5 [40.5 to 48.6]	2.1 [−3.5 to 7.7]	0.454
SF-36 subscales^c^ after 8 weeks					
General health	48	58.3 [53.1 to 63.4]	51.5 [46.4 to 56.6]	6.8 [−0.6 to 14.1]	0.069
Mental health	48	65.1 [60.8 to 69.3]	64.1 [59.8 to 68.4]	1.0 [−5.1 to 7.0]	0.751
Bodily pain	48	49.3 [43.6 to 55.0]	45.6 [39.9 to 51.3]	3.6 [−4.4 to 11.7]	0.369
Physical functioning	48	74.7 [70.5 to 79.0]	74.2 [69.9 to 78.5]	0.5 [−5.5 to 6.6]	0.858
Role-emotional	48	69.4 [58.1 to 80.7]	73.7 [62.3 to 85.0]	−4.3 [−20.3 to 11.8]	0.593
Role-physical	48	68.6 [54.6 to 82.6]	51.2 [37.2 to 65.2]	17.4 [−2.4 to 37.2]	0.083
Social functioning	48	73.2 [66.4 to 79.9]	74.2 [67.5 to 81.0]	−1.1 [−10.7 to 8.5]	0.821
Vitality	48	47.8 [44.2 to 51.5]	46.7 [43.1 to 50.3]	1.1 [−4.0 to 6.2]	0.666
SF-36 subscales^c^ after 12 weeks					
General health	47	61.0 [55.6 to 66.4]	51.0 [45.5 to 56.5]	10.0 [2.2 to 17.8]	0.013
Mental health	47	67.2 [61.0 to 73.3]	62.2 [56.0 to 68.5]	4.9 [−3.8 to 13.7]	0.263
Bodily pain	47	53.0 [45.5 to 60.4]	42.9 [35.3 to 50.5]	10.1 [−0.6 to 20.8]	0.063
Physical functioning	47	73.8 [69.2 to 78.4]	72.3 [67.6 to 77.0]	1.5 [−5.1 to 8.1]	0.644
Role-emotional	47	65.8 [51.4 to 80.2]	61.8 [47.1 to 76.5]	4.0 [−16.5 to 24.6]	0.696
Role-physical	47	63.4 [48.8 to 77.9]	50.2 [35.3 to 65.1]	13.2 [−7.7 to 34.0]	0.209
Social functioning	47	75.4 [67.0 to 83.7]	70.8 [62.3 to 79.4]	4.5 [−7.5 to 16.5]	0.450
Vitality	47	52.0 [47.3 to 56.7]	45.1 [40.3 to 49.9]	7.0 [0.2 to 13.7]	0.043

^a^ Results adjusted for respective baseline value.

^b^ Lower values indicate better status.

^c^ Higher values indicate better status.

CI, confidence interval; FFbH-R, Hannover functional questionnaire backache; MWP, mindful walking program; *n* = number for respective available data; PSS-14, 14-item Cohens' perceived stress scale; SF-36, short-form-36 health survey; VAS, visual analog scale; MCID, minimal clinically important difference.

Neither imputation of missing endpoint values nor adjustment for additional baseline variables or repeating the analysis with the PP (*n* = 44) produced a relevant change in the results with the primary outcome. We found no significant or clinically relevant mean differences between the MWP group and the CG for back function (FFbH-R), 2.2 [−4.2 to 8.6], *p*-value = 0.493; for stress (PSS-14), −1.6 [−4.8 to 1.6], *p*-value = 0.326; for health-related quality of life (SF-36 physical component scale), 3.5 [−0.2 to 7.3], *p*-value = 0.066; and health-related quality of life (SF-36 mental component scale), −1.2 [−5.5 to 3.0], *p*-value = 0.567. After 8 weeks, out of 48 patients, 15 patients in the MWP group (62.5%) and one patient in the CG (4.2%) reported an improvement in low back pain (pain “slightly reduced” or “significantly reduced”).

Within the first 8 weeks, the intake of rescue medication (paracetamol) was comparable between groups, with an MWP group mean ± SD of 5.4 ± 13.1 (range: 0–48) and a CG mean of 5.4 ± 8.7 (range: 0–30). During this time, 16 (55.2%) patients in the MWP group and 14 (53.9%) patients in the CG took no analgesic medication. Very few patients reported the use of analgesics other than the recommended rescue medication paracetamol. In the MWP group, the only additional analgesic was diclofenac taken by one patient in 1 week. In the CG, ibuprofen was taken by one patient in 4 weeks.

The total costs for analgesics used in the first 8 weeks were 78.66 (Euro, MWP group) versus 66.69 Euro (CG), with an average cost per week of 6.05 ± 9.01 Euro vs. 5.15 ± 4.46 Euro, respectively, *p*-value = 0.570.

During the first 8 weeks, no patient in the CG, but three patients in the MWP group, reported adverse events (*n* = 1 pneumonia with suspected pertussis, *n* = 1 toe injury (hemotoma) during a women's run competition, and *n* = 1 toe injury during weekend cycling), all not causally related to the intervention.

**FIG. 2.**
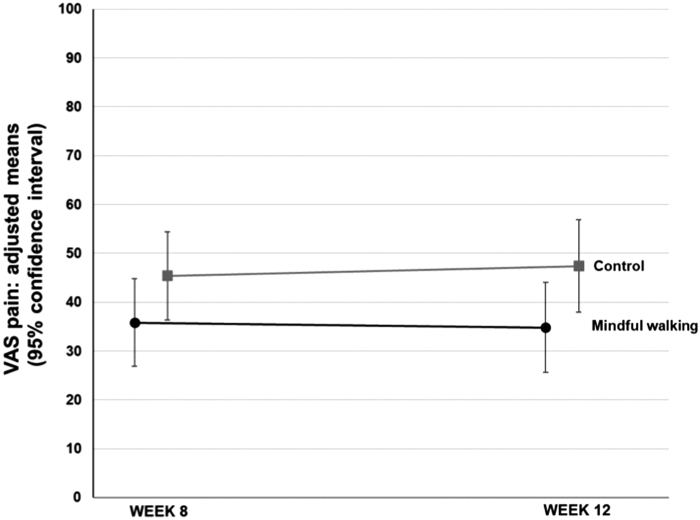
Adjusted means and 95% confidence intervals of the last average pain intensity in the last week (VAS) at 8 and 12 weeks with *p*-values comparing mindful walking with no intervention (lower values indicating less pain). VAS, visual analog scale.

Between weeks 8 and 12, 19 (65.5%) patients in the MWP group continued to exercise for themselves. After 12 weeks, the intergroup difference in VAS pain increased to −12.6 [−25.8 to 0.6], *p*-value = 0.061 (Fig. 2). The between-group differences in back function assessed by the FFbH-R, stress assessed by the PSS-14, and health-related quality of life assessed by the SF-36 physical component scale (but not by the SF-36 mental component scale) tended to favor the MWP compared with the control conditions but failed to reach clinical relevance.

## Discussion

In this single-center, open, two-armed randomized controlled clinical trial in CLBP patients, eight weekly MWP sessions did not result in statistically significant or clinically relevant group differences in the mean low back pain intensity in comparison to no mindful walking. In addition, secondary outcomes showed no clinically relevant differences between the study groups after 8 weeks. After 12 weeks, the intergroup difference in the VAS score for pain broadened, favoring mindful walking, but was still not clinically relevant. The analgesic intake was comparable in both groups; however, analgesic costs were slightly higher for patients in the MWP group than for controls. No adverse events were associated with the MWP.

The strengths of this RCT were the use of a previously tested intervention program, the comprehensive range of validated patient-reported outcomes, and the follow-up 4 weeks after the end of the therapy. The rather homogeneous study population regarding pain intensity due to the inclusion criteria and the standardized intervention program augment the internal validity of the study. The external validity was augmented by implementing three therapists deriving from three different professions.

This study has limitations. This RCT employed a single-center setting and included a high percentage of women. These factors limit the generalizability of our results. We excluded patients with severe organic, psychological, or psychiatric disorders. However, we did not systematically assess psychiatric comorbidities. Psychiatric comorbidities, such as depressive disorders, may have an impact on pain, stress, and function in patients with CLBP and may have influenced the results of our study.

The frequency of self-applications and non-drug adjunctive therapies was not determined, which may have contributed to a decrease in the group difference in the primary and secondary outcome parameters as the participants in the CG may also have started mindful self-applications. Despite giving instructions, mindfulness was not controlled in this study. Suitable instruments for measuring mindfulness are available.^38–42^ The study design had several potential sources of bias: Patients in the MWP group received time and attention that patients in the CG did not receive, and we did not include blinded outcome assessments.

Considering these factors, tendencies for treatment effects may have been overestimated. However, recently, the relevance of blinding in RCTs has been discussed, as a meta-epidemiological study found no evidence for an average difference in estimated treatment effects between trials with and without blinded patients, health care providers, or outcome assessors.^43^ However, the lack of blinding might have an impact on the results in our population, as the patients expected more positive results if randomized to the MWP group.

We used mostly validated outcomes. The assessment of analgesic use from patient diaries was not validated but standardized because analgesics might cause relevant life-threatening side effects,^9–12^ and reduction in their use is highly relevant to the patients. In our opinion, the evaluation of analgesics is important for the interpretation of study results, and it was performed earlier in trials on CLBP and chronic neck pain.^44–50^ However, the obtained results must be interpreted with caution.

To facilitate compliance in the MWP group, we chose to provide eight sessions of MWP, with a pragmatic duration of ∼60 min each. Longer MWP treatments, such as eight weekly 1.5- to 2.5-h sessions, as performed in MBSR trials in patients with low back pain,^19^ might have provided stronger effects on pain intensity. To maintain the pragmatic advantage of the shorter 60-min interventions, the time available for individual mindful walking could be progressively significantly increased. For example, mindful walking could last 30 min from week 5. Further, a more intensive exercise component could have had a stronger effect on pain intensity, as exercise is an important and potent component of the multimodal therapy regimen for CLBP.^6,7^

We instructed patients in the MWP group to self-exercise between sessions but did not assess adherence to the self-exercises. Such an assessment could have been a motivational factor and possibly resulted in a higher treatment effect.

We used the VAS for pain intensity measurement as the primary outcome, because pain is relevant to patients. The VAS assessments of pain have been validated, widely used, and established in our research groups; are easy to use; and require less than 1 min to complete.^26,29,44,47,51–56^ However, recently, their validity has been questioned.^57^ In patients with CLBP, the between-group difference was not statistically significant in our study. This might be due to various factors. First, we might have overestimated the treatment effects of our MWP, and in a larger study population, the treatment effects could have become more evident.

For low back pain (any duration), a meta-analysis included four RCTs comparing MBSR (171 patients) with routine care (155 patients)^19^ and found a statistically significant but not clinically relevant improvement in pain intensity (numeric rating scale, range: 0–10, MCID: 1.5) at short-term follow-up (mean difference: 0.96 [95% CI 1.64–0.34]). We found a comparable mean difference between groups after 8 weeks with −9.6 [−22.3 to 3.1] on a VAS (0–100 mm) with our rather easy-to-apply MWP. However, the mentioned meta-analysis^19^ found no statistically significant or clinically important group differences (mean difference: 0.90 [7.66–5.86]) in pain intensity at long-term follow-up (6 months after randomization) in two RCTs.^19^

In contrast, we found an indication for a slightly larger intergroup difference at 12 weeks after randomization in our study. This might be partly due to the high percentage of patients who continued to walk after 8 weeks and to the shorter observation time compared with the mentioned meta-analysis. A previous systematic review on “acceptance- and mindfulness-based interventions” included 25 RCTs (1285 patients) with various chronic pain conditions. It reported smaller beneficial effects for pain intensity (range: 0–10) with a pooled standard mean difference of 0.24 [0.06–0.42].^17^

Michalsen et al. conducted an RCT involving 68 patients with CLBP and found no differences in the primary outcome of VAS pain in a focused meditation versus self-care exercise comparison.^58^ Regarding back function, measured by the German tool FFbH-R, we found hints for a small nonclinically relevant effect in the MWP group compared with the CG after 8 weeks, but this difference diminished after 12 weeks. In contrast, the meta-analysis mentioned earlier^19^ reported two studies that investigated MBSR compared with routine care in patients with low back pain with statistically significant but clinically irrelevant improvement in physical functioning, and these benefits were not observed at the follow-up.

Further, the authors^19^ found no statistically significant or clinically relevant benefit of MBSR compared with active controls in short- or long-term analyses on health-related quality of life. This is in line with our results for health-related quality of life and stress. However, in our previous randomized clinical trial in patients with stress symptoms,^27^ a less physically active MWP, compared with control conditions, resulted in a statistically significant reduction in perceived stress assessed with the PSS-14. Further, a more recent trial on MBSR in patients with CLBP (involving 17 sequentially sampled patients in the intervention condition and 11 patients in the waiting-list control condition) reported between-group medium-to-large effect sizes with a pre–post comparison of pain severity and quality of life.^59^

## Conclusion

Practicing mindful walking in patients with low back pain did not result in relevant improvements in pain, stress, or back function compared with no intervention. Future studies should consider intensifying the mindful walking intervention and including long-term follow-up.
